# P-1763. Disease burden of carbapenem-resistant Enterobacterales (CRE) infections in Korea

**DOI:** 10.1093/ofid/ofaf695.1934

**Published:** 2026-01-11

**Authors:** Chan Mi Lee, Eunyoung Lee, Shinwon Lee, Sun Young Cho, Pyoeng Gyun Choe, Raeseok Lee, Jiwon Jung, Nam Su Ku, Young Keun Kim, Hee Jung Choi, Jeong-Han Kim, Chung-Jong Kim, Yee Gyung Kwak, Kyung-Hwa Park, Sung un Shin, Yeon Sook Kim, Shinhye Cheon, Se Yoon Park, Jeonghoon Ahn, Kyoung-Ho Song

**Affiliations:** Seoul National University College of Medicine, Seoul, Seoul-t'ukpyolsi, Republic of Korea; Seoul National University College of Medicine, Seoul, Seoul-t'ukpyolsi, Republic of Korea; Division of Infectious Disease, Department of Internal Medicine, Pusan National University Hospital, Seo-gu, Pusan-jikhalsi, Republic of Korea; Samsung Medical Center, Seoul, Korea, Seoul, Seoul-t'ukpyolsi, Republic of Korea; Seoul National University College of Medicine, Seoul, Seoul-t'ukpyolsi, Republic of Korea; Seoul St. Mary's Hospital, College of Medicine, The Catholic University of Korea, Seocho-gu, Seoul-t'ukpyolsi, Republic of Korea; Asan Medical Center, Seoul, Seoul-t'ukpyolsi, Republic of Korea; Division of Infectious Diseases, Department of Internal Medicine, Yonsei University College of Medicine, Seoul, Seoul-t'ukpyolsi, Republic of Korea; Yonsei University Wonju College of Medicine, Wonju, Kangwon-do, Republic of Korea; Ewha Womans University College of Medicine, Seoul, Seoul-t'ukpyolsi, Republic of Korea; Ewha Woman University College of Medicine Mokdong Hospital, Department of Internal Medicine, Yangcheon-gu, Seoul-t'ukpyolsi, Republic of Korea; Ewha Womans University College of Medicine, Seoul, Seoul-t'ukpyolsi, Republic of Korea; Inje University Ilsan Paik Hospital, Ilsan, Kyonggi-do, Republic of Korea; Chonnam National University Medical School, GwangJu, Kwangju-jikhalsi, Republic of Korea; Chonnam National University hospital, Kangju, Kwangju-jikhalsi, Republic of Korea; Chungnam National University School of Medicine, Daejeon, Ch'ungch'ong-namdo, Republic of Korea; Chungnam National University Hospital, Daejeon, Ch'ungch'ong-namdo, Republic of Korea; Division of Infectious Diseases, Department of Internal Medicine, Soonchunhyang University Seoul Hospital, Seoul, Seoul-t'ukpyolsi, Republic of Korea; Ewha Womans University, Seoul, Seoul-t'ukpyolsi, Republic of Korea; Seoul National University Bundang Hospital, Seoul National University College of Medicine, Seoungnam-si, Kyonggi-do, Republic of Korea

## Abstract

**Background:**

Carbapenem-resistant *Enterobacterales* (CRE) pose a significant public health threat. While CRE isolation from clinical specimens is a mandatory national notifiable event in Korea, current surveillance systems do not differentiate between colonization and infection, hindering accurate estimation of the disease burden. This study aimed to assess the clinical and socio-economic burden of CRE infections in Korea.

**Figure:**
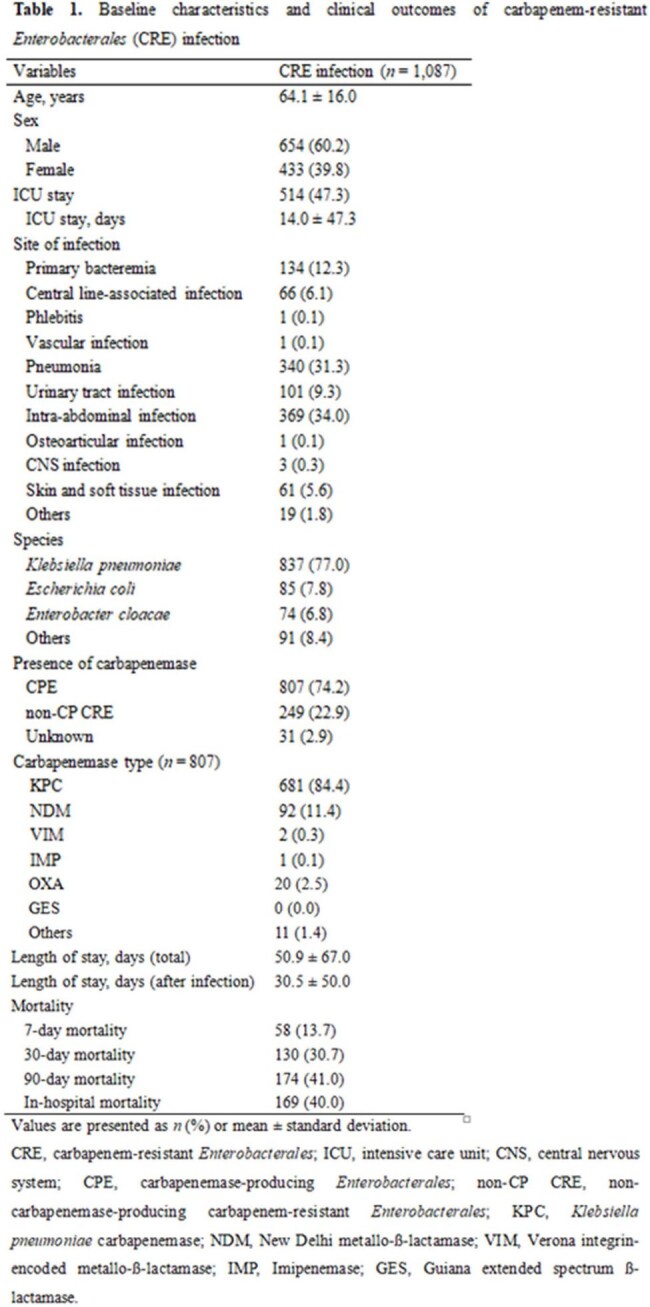

**Figure:**
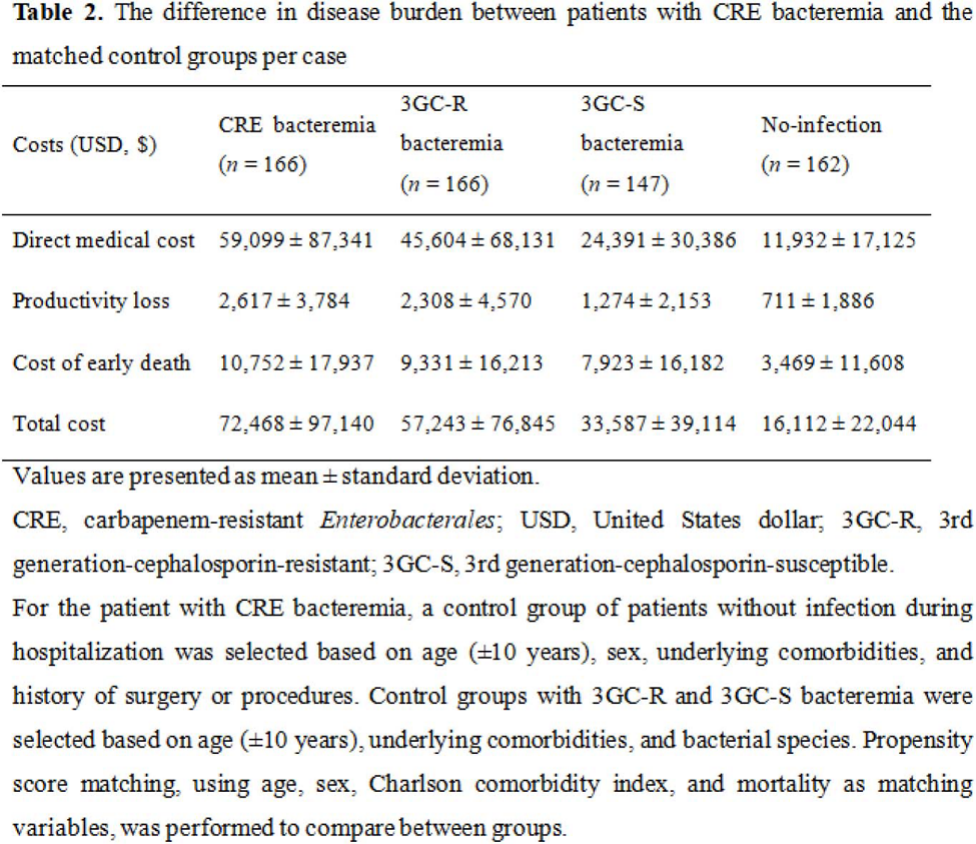

**Methods:**

A retrospective study was conducted across fifteen geographically diverse medical institutions. Medical records from all cases with CRE-positive clinical specimens in 2022 were reviewed and classified as “CRE-infected” or “colonized.” For the “CRE-infected” cohort, clinical data and medical costs were collected. A predictive logistic regression model was developed using sex, age, presence of bacteremia, and bacterial species to estimate the nationwide CRE infections. For cases of CRE bacteremia, matched control groups (no-infection, 3rd generation-cephalosporin-susceptible [3GC-S], and 3GC-resistant [3GC-R] infections) were used to assess the incremental burden of CRE.

**Results:**

Of 4,848 reported CRE cases, accounting for 15.9% of the 30,548 reported in Korea, 1,087 (22.4%) were classified as infections, including 586 bacteremic cases. Intra-abdominal infection (34.0%) and pneumonia (31.3%) were the most common infection sites. *Klebsiella pneumoniae* was the most frequent pathogen (77.0%) of CRE infections. Carbapenemase-producing *Enterobacterales* accounted for 74.2%, with KPC (84.4%) being the most common type. The 30-day and 90-day mortality rates were 29.3% and 40.8%, respectively. Nationwide, 7,117 CRE infections were estimated by the predictive logistic regression model, including 1,897 bacteremia. The total cost per CRE infection was estimated at $78,071. The economic burden of CRE infections in 2022 was estimated at $517,610,261, exceeding that of no-infection, 3GC-S, and 3GC-R infections by $108,355,500 to $401,087,500.

**Conclusion:**

This study confirmed the profound clinical and socioeconomic burden of CRE infections in Korea. Considering the rapidly increasing number of reported CRE cases even after 2022, it is urgent to prepare corresponding measures against CRE infections.

**Disclosures:**

Kyoung-Ho Song, MD, PhD, PhAST Corp.: Advisor/Consultant|PhAST Corp.: Grant/Research Support

